# Solvatochromic, spectroscopic, DFT calculations, antimicrobial and docking studies of new Fe(III), Co(II), and Ni(II) chelates containing 1,2,4-triazine

**DOI:** 10.1038/s41598-026-48416-3

**Published:** 2026-04-25

**Authors:** Ebtesam M. Abdelrhman, Fatma Samy, Omima M.I. Adly, Mona Boshra, Mohamed F. Eid, Nesma Salah

**Affiliations:** Chemistry Department, Faculty of Education, Ain Shams University, Cairo, Egypt

**Keywords:** 1,2,4-triazine, Hydrazone, Chelates, Antimicrobial activity, DFT, NLO, 1HNJ, Biochemistry, Chemistry

## Abstract

This study presents the synthesis and characterization of three novel metal chelates: **Ni(DTHMN)** (**1**), **Co(DTHMN)** (**2**), and **Fe(DTHMN)** (**3**). Comprehensive analytical techniques, including elemental analysis, infrared (IR) and electronic (UV-Vis) spectroscopy, thermal analysis, molar conductivity, and magnetic susceptibility measurements, were employed to investigate their structural and electronic properties. The findings indicate that the ligand **DTHMN** acts as a tridentate donor, coordinating through the hydroxyl group (O–H), azomethine nitrogen (C = N), and triazine nitrogen atoms, forming mononuclear chelates with tetrahedral or octahedral geometries. X-ray diffraction (XRD) and transmission electron microscopy (TEM) analyses confirmed that the **Ni(DTHMN)** chelate adopts a nano-spherical to cubic morphology. All synthesized chelates exhibit luminescence behavior, suggesting potential utility in photoactive materials. Solvatochromic shifts in absorption and fluorescence spectra were analyzed to estimate ground-state (µ_g_) and excited-state (µ_e_) dipole moments. These were determined using Reichardt’s solvent polarity parameter (E_T_^N^) and multiple empirical functions, including the Bilot–Kawski, Lippert–Mataga, Bakhshiev, and Kawski–Chamma–Viallet models. A notable increase in dipole moment upon excitation suggests enhanced stabilization of the excited state in polar solvents, particularly involving n–π* transitions. Density Functional Theory (DFT) calculations, performed using the B3LYP/GENECP method with a 6-311G(d, p) basis set for non-metal atoms and SDD for metal atoms, supported experimental data and provided insights into optimized structural parameters. Further computational analyses included molecular electrostatic potential (MEP) mapping and nonlinear optical (NLO) property evaluation, revealing charge distribution and optical characteristics. The antimicrobial activity of the complexes was tested against a panel of Gram-positive, Gram-negative bacteria, and fungi, showing promising efficacy. Finally, molecular docking studies were conducted to investigate the binding interactions of the chelates with the FabH–CoA enzyme complex (PDB ID: 1HNJ), providing insight into their potential as bioactive agents.

**Supplementary Information:**

The online version contains supplementary material available at 10.1038/s41598-026-48416-3.

## Introduction

Research on hydrazones with N, S, and O heterocycles, such as 1,2,4-triazines, has acquired considerable interest in recent years^1,2^. The azomethine linkage and additional donor sites in 1,2,4-triazine moieties were proposed to enhance their flexibility and versatility in terms of biological, pharmacological, and medicinal properties^3,4^. Triazine derivatives have long been employed as herbicides or pesticide components in agriculture, as complexing agents in analytical chemistry, and as multi-step redox systems in electrochemistry^5^. They possess antitubercular^6^, antimycotic^7^, antimicrobial^8^, antipyretic^9^, and antihypertensive properties^10^. The presence of the -OH group increases the reactivity of hydrazones exponentially, especially when the -OH group is present at the -ortho position^11^. Hydrazones derived from naphthaldehyde are highly effective and exhibit a range of pharmacological activities, including antiproliferative^12^, antibacterial^13^, and urease inhibitory effects^14^, as well as the ability to bind to and cleave DNA^15^. Also, medications with naphthaldehyde moieties are well-known chemotherapeutic medicines that have been used to treat cancer and tumors^16,17^. In medicinal chemistry, heterocyclic hydrazone metal chelates are essential^18^. The ability of several transition metal chelates to cleave DNA and to function as catalytic, antioxidant, antitumoral, antibacterial, anti-inflammatory, antiproliferative, antiviral, antimalarial, and antimycotic substances has been studied^19–22^. In bioinorganic chemistry, the Co(II) and Ni(II) chelates have significant roles in DNA breakage, antioxidant, antibacterial, antifungal, and anticancer properties^23,24^. Co(II) and Ni(II) chelates exhibit beneficial properties such as efficient cellular membrane permeability, minimal cytotoxicity, and broad-spectrum therapeutic potential across various pathological conditions^25,26^. Due to our interest in compounds based on 1,2,4-triazines^27–33^. This study reports the successful synthesis and detailed characterization of three novel metal chelates with a tridentate ligand (**DTHMN**), offering valuable insights into coordination chemistry. Comprehensive analyses, including IR, UV-Vis, thermal analysis, XRD, TEM, conductivity, and magnetic measurements, reveal key structural and electronic features. DFT calculations support experimental results by providing insights into optimized geometries, electrostatic potentials, and nonlinear optical properties, suggesting potential in optoelectronics. All chelates exhibit luminescent and solvatochromic behavior, with dipole moment studies indicating excited-state stabilization in polar solvents, promising for fluorescence-based sensors. Antimicrobial assays reveal activity against various strains, supported by molecular docking with the FabH–CoA enzyme (PDB ID: 1HNJ), indicating therapeutic potential. Overall, this work integrates synthesis, computational modeling, and biological evaluation to advance multifunctional materials for applications in sensing, medicine, and optoelectronics.

## Experimental

### Materials

All materials used in this study were of analytical reagent (AR) grade. All chemical compounds, solvents, and reagents, including Ni(II) and Co(II) metal salts as acetates and Fe(III) as nitrate salt, thiosemicarbazide, benzil, glacial acetic acid, 2-hydroxy-1-naphthaldehyde, LiOH.H_2_O, and 100% hydrazine hydrate, were procured from Sigma-Aldrich and employed without further purification.

### Synthesis of hydrazone ligand (DTHMN)

The hydrazone ligand **DTHMN** was synthesized by the procedure described in the literature and is documented in the **Supplementary File Data S1**^33^.

### Synthesis of metal chelates

**Scheme 1:**
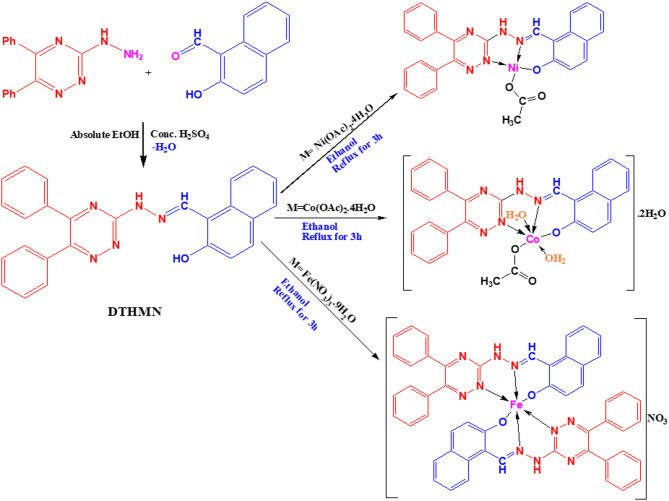
Preparation of **M(DTHMN)** chelates.

An ethanolic solution of (**DTHMN**) (0.5 g, 1.20 mmol) and LiOH.H_2_O (0.1 g, 2.40 mmol) dissolved in water (10 mL) was added gradually with continuous stirring to metal salts namely, Fe(NO_3_)_3_.9H_2_O, Co(OAc)_2_.4H_2_O and Ni(OAc)_2_.4H_2_O (1.20 mmol) dissolved in ethanol (20 mL). The mixture of an ethanolic solution of metal ions and a hot ethanolic solution of deprotonated ligand by LiOH in a molar ratio of 1:1:1 (**DTHMN**: LiOH: M) was heated under reflux for six hours. The resulting metal chelates (Scheme 1) were isolated by filtration and thoroughly washed several times with a 50% (v/v) ethanol-water solution to ensure the complete removal of unreacted starting materials, followed by a final wash with diethyl ether. The metal complexes were stored in a desiccator.

### Measurements

Full information on all instruments employed in this study is presented in the supplemental materials (Supplementary File Data S2).

### Antimicrobial activity

#### Agar well diffusion method

The biological activity of the chelates was evaluated using the standard disc-agar diffusion method^34^. The organisms consist of three different types: fungal strains (*Candida albicans*), Gram-negative bacteria (*Escherichia coli*), and Gram-positive bacteria (*Staphylococcus aureus)*. The standards were the antibiotics cephalothin and chloramphenicol for Gram-negative and Gram-positive bacteria, and cycloheximide for fungi.

#### Minimum Inhibitory Concentration (MIC) assay

A series of **M(DTHMN)** chelates concentrations was assessed for their antimicrobial efficacy against *Staphylococcus aureus*, *Escherichia coli*, and *Candida albicans*. The minimum inhibitory concentration (MIC) was defined as the lowest concentration at which total inhibition of microbial growth was observed, as evidenced by the complete absence of visible colonies^35^.

### Theoretical calculations

#### Molecular modeling

Geometry optimizations of the newly synthesized metal chelate in both ground and excited electronic states were conducted using the *Gaussian 09* software package^36^. Utilizing DFT/TD-DFT/GENECP methods for calculations of their structural parameters. The metal ions were described using Effective Core Potentials (GENECP) basis set with SDD, which is a keyword used in the basis set specification part of the input file. In contrast, the 6-311G(d, p) basis set was used for non-metal atoms, including hydrogen, carbon, nitrogen, and oxygen, to incorporate polarization functions and enhance precision in describing electronic distributions^37^. The use of mixed basis sets was carefully selected according to the atomic characteristics of the studied metal centers. Specifically, GENECP and SDD were applied as effective core potentials (ECPs) for heavy atoms to reduce computational cost while maintaining accuracy. This combination ensures consistency, computational efficiency, and reliable prediction of molecular geometries, energies, and electronic properties across all complexes. The optimized molecular structures were visualized using the GaussView 5.0 package^38^. Molecular electrostatic potential (MEP) maps were generated to depict the spatial distribution of electrostatic potential across the molecular surface. The frontier molecular orbitals (FMOs) play a pivotal role in defining the electronic structure and optical behavior of the molecule^38^, with the energy difference between the HOMO and LUMO serving as a fundamental indicator of molecular stability and reactivity^39^. The nonlinear optical (NLO) properties of the investigated chelates were evaluated through density functional theory (DFT) calculations. Since *Gaussian 09* reports polarizability and hyperpolarizability in atomic units.

#### Molecular docking

Auto Dock has been utilized to perform molecular docking of the synthesized chelates^40^. Molecular docking was employed as a crucial method to explore the complex binding interactions of the studied compounds with the *E. coli* FabH–CoA complex (PDB ID: 1HNJ)^41^. The FabH receptor is targeted to assess the potential of these molecules as antimicrobial agents, given FabH’s role in the biosynthesis of fatty acids^41^. The three-dimensional structure of the target receptor was obtained from the Protein Data Bank (https://www.rcsb.org/structure/1HNJ). Before docking, the receptor structure (PDB ID: 1HNJ) was prepared by removing water molecules, ligands, and heteroatoms, followed by the addition of polar hydrogen atoms and Kollman charges^42^. The synthesized compounds were initially sketched using ChemDraw, and their energies were minimized and then converted to PDBQT format. A grid box was defined for the 1HNJ receptor with coordinates (X = 26.747, Y = 13.337, Z = 33.312) and dimensions (X = 15.302 Å, Y = 21.817 Å, Z = 14.604 Å). The binding affinities of the compounds were subsequently evaluated based on their binding energies (kcal/mol)^43^. This computational study provides a detailed examination of the interactions between ligands and the FabH receptor, offering critical insights into their pharmacological properties and potential therapeutic applications.

## Results and discussion

The isolated metal chelates, [Ni(**DTHMN**)(OAc)], [Co(**DTHMN**)(OAc)(H_2_O)_2_].2H_2_O, and [Fe(**DTHMN**)_2_]NO_3_, are highly thermally stable due to their high melting temperatures (over 300 °C) (Table 1). These chelates are colored, non-hygroscopic, remain stable under ambient conditions, and exhibit low solubility in water and in most organic solvents. To elucidate their predicted structures, the metal chelates were examined using various spectroscopic techniques, along with magnetic susceptibility and conductivity measurements, as well as elemental and thermal analyses.

**Table 1:** Analytical and physical data of metal chelates.

Complex	M. F. [F. Wt]	Color	Yield (%)	Elemental analysis, % Found/(Calc.) C H *N* M			
[Ni(**DTHMN**)(OAc)] **(1)**	C_28_H_21_N_5_O_3_Ni [534.19]	Light brown	0.54 g (84.8)	62.60 (62.95)	3.46 (3.96)	13.60 (13.11)	10.44 (10.98)
[Co(**DTHMN**)(OAc)(H_2_O)_2_].2H_2_O **(2)**	C_28_H_29_N_5_O_7_Co [606.49]	Brown	0.39 g (53.60)	56.09 (55.54)	4.70 (4.82)	11.34 (11.54)	9.85 (9.72)
[Fe(**DTHMN**)_2_]NO_3_ **(3)**	C_52_H_36_N_11_O_5_Fe [950.76]	Black	0.68 g (59.60)	65.16 (65.69)	3.65 (3.81)	15.89 (16.20)	5.39 (5.87)

### IR spectra

IR spectra of the synthesized compounds were recorded using KBr discs over the spectral range of 4000–400 cm⁻¹. Table 2 summarizes the principal vibrational frequencies along with their corresponding band assignments. Comparative analysis of the IR spectra of the free **DTHMN** ligand and its metal chelates was conducted to confirm coordination through changes in characteristic absorption bands. The free ligand **DTHMN** exhibited prominent IR bands at 1617, 1518, 1464, and 1293 cm^− 1^, which were attributed to ν(C=N_azomethine_), ν(C=N_triazine_), ν(N=N_triazine_), and ν(N–N_triazine_) stretching vibrations, respectively^33^. Upon complexation with Ni(II), Co(II), and Fe(III) ions, these bands shifted to lower frequencies, specifically to the regions (1598–1600) cm^− 1^ ν(C=N_azomethine_), (1489–1516) cm^− 1^ ν(C=N_triazine_), (1407–1421) cm^− 1^ ν(N=N_triazine_), and (1266–1283) cm⁻¹ ν(N–N_triazine_). These shifts are revealing of coordination through the azomethine and triazine nitrogen atoms, as well as through the deprotonated hydroxyl group, confirming the ligand’s positions in chelation^44,45^. Additionally, broad absorption bands observed in the 3169–3461 cm^− 1^ range in all metal chelates were assigned to stretching vibrations of coordinated OH and/or NH groups^44^. The chelating character of the acetate moiety was confirmed by the medium bands (1444–1450) and 1250 cm^− 1^, which are assigned to ν_as_(COO^−^) and ν_s_(COO^−^), respectively, for the acetate of **Ni(DTHMN**) and **Co(DTHMN)** chelates^44,46^. The monodentate state of the acetate anion can be observed through a significant comparison between the two bands. On the other hand, **Fe(DTHMN)** exhibited bands at 1355 and 825 cm^− 1^, confirming the presence of the ionic NO_3_^−^ group^44,46^. The newly assigned bands at 545–560 and 419–485 cm^− 1^, attributed to ν(M–O) and ν(M–N), respectively, support the proposed chelation mechanism^47,48^. Moreover, the negative slope of νM-O *versus* C = N, νM-O/cm^− 1^ = 49,032–30.32 ν_C=N_/cm^− 1^, *r* = 0.99, *n* = 3 points demonstrate that strong contact of C-O with metal ions was accompanied by a large amount of back donation to the azomethine group, which improves its association with metal ions (appeared at a lower frequency). Furthermore, M-N/cm^− 1^ = 2.9113 + 23.20 Δν_C=N_/cm^− 1^, *r* = 0.99, *n =* 3 points. The positive slope reveals that the strong M-N bond is accompanied by a higher shift of C = N to a lower frequency.

**Table 2:** Characteristic IR spectral data of **DTHMN** ligand^33^ and its metal chelates.

Compound		IR Spectra (cm^− 1^)						
	ν(OH)/ ν(*N*-H)	ν(C = *N*) Azomethine	ν(C = *N*) Triazine	ν(*N* = *N*) Triazine	ν(*N*-*N*) Triazine	ν(M-*N*)	ν(M-O)	Other bands
DTHMN	3473, 3226	1617	1518	1464	1293	------	-----	3052 ν(CH _aromatic_), 2976 ν(CH_aliphatic_)
Ni(DTHMN)	3461	1598	1489	1407	1283	419	555	1450 ν_as_(COO^−^),1250 ν_s_(COO^−^); monodentate (AcO^−^)
Co(DTHMN)	3420	1600	1516	1416	1283	485	560	1444ν_as_(COO^−^), 1250ν_s_(COO^−^); monodenatae (AcO^−^)
Fe(DTHMN)	3383, 3169	1600	1516	1421	1266	425	545	1355, 825; ν(NO_3_^−^) (ionic)

### Electronic spectra and magnetic moment measurements

**Fig. 1:**
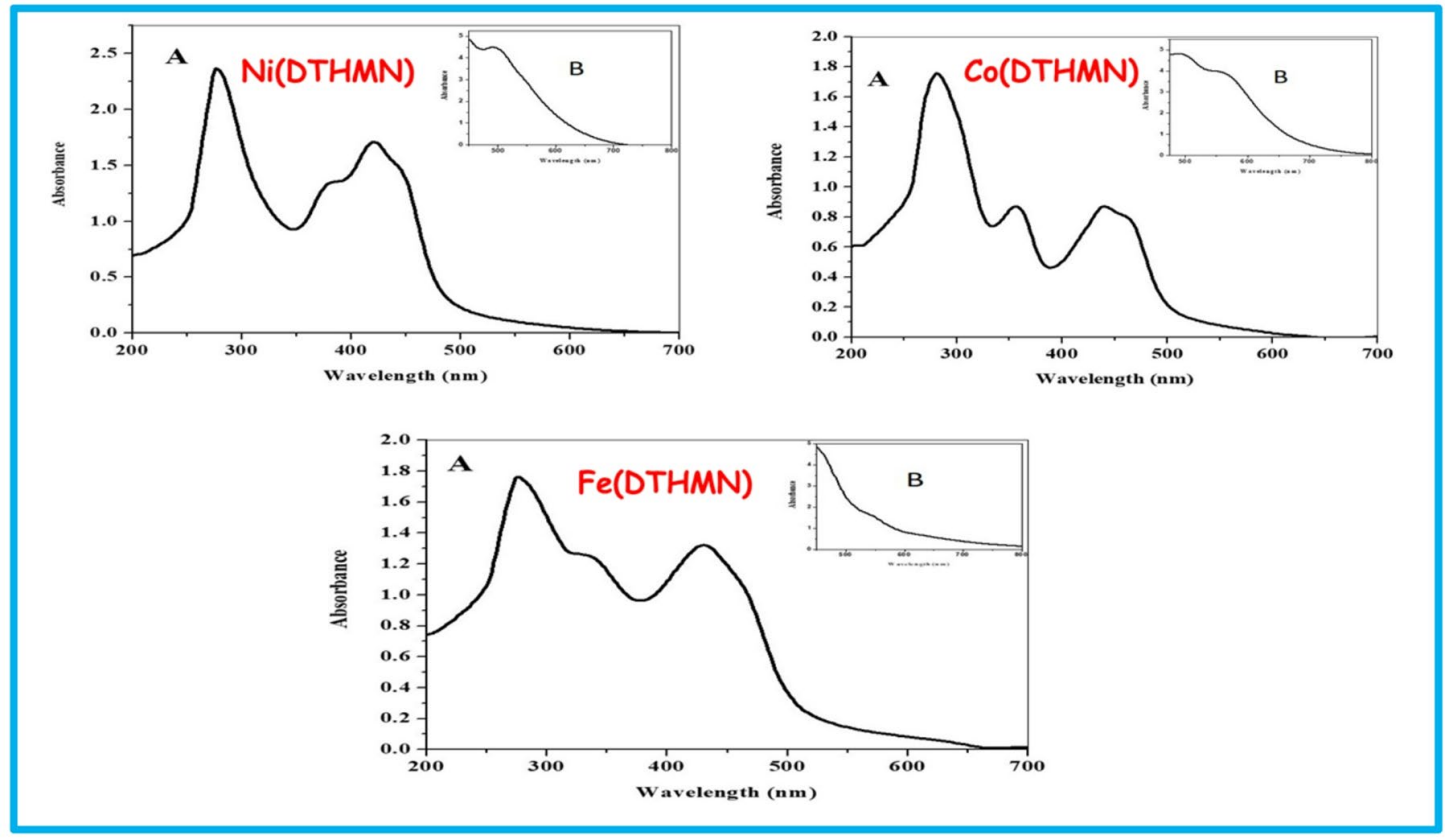
UV-Vis spectra of **M(DTHMN)** chelates.

The UV-Vis spectra were systematically evaluated to elucidate the coordination geometry and identify the intra-ligand (π→π* and n→π*), metal-to-ligand charge transfer (MLCT), and d-d electronic transitions of **DTHMN** and its corresponding metal chelates. Spectroscopic measurements were conducted in a DMF solution and by reflectance techniques. Table 3 summarizes the electronic spectral as well as magnetic susceptibility data. Although the distinctive absorption bands of the free ligand **DTHMN** were still present during complexation, they changed in position, intensity, and spectral profile, which is evidence of coordination with metal centers. Moreover, new intense absorption bands emerged within the visible region, attributable to d-d transitions associated with the respective metal ions, which further confirm successful complex formation and support the proposed geometries. The electronic spectrum of the **Ni(DTHMN)** chelate (Fig. 1) exhibited absorption bands at 276, 380,421, and 500 nm. In addition to the ligand-associated absorption bands observed at 276 nm (π→π*) and 380 nm (n→π*), a peak centered at 421 nm is attributed to ligand-to-metal (L→M) charge transfer (CT) transitions. The d-d- transition band observed at 500 nm, which may be ascribed to ^3^T_1_→^3^T_1_(P) (ν_3_) transition in a tetrahedral geometry^30^. The effective magnetic moment value at 298 K of **Ni(DTHMN)** chelate is 3.50 B.M., which is in the expected range (3.4–3.8 B.M.) of the tetrahedral Ni(II) in the chelate geometry^49,50^. The electronic spectrum of the **Co(DTHMN)** chelate (Fig. 1) showed absorption bands at 497 and 564 nm corresponding to ^4^T_1g_(F)→^4^T_1g_(p) and ^4^T_1g_(F)→^4^A_2g_(F) transitions, which is consistent with the octahedral geometry of the metal chelates^51^. Ligand-positioned absorption bands are detected at 282 nm and 356 nm, corresponding to π→π* and n→π* electronic transitions, respectively. Furthermore, a pronounced charge transfer (CT) band is observed at 441 nm. The effective magnetic moment at 298 K of the **Co(DTHMN)** is 4.96 B.M., which falls in the 4.7–5.2 B.M. range expected for octahedral geometry^51^. The electronic spectrum of the **Fe(DTHMN)** (Fig. 1) showed an absorption band at 276, 338, 430, and 548 nm. The first two bands at 276 nm (π→π*) and 338 nm (n→π*) fall within the ligand-centered region, whereas the latter two bands are at 430 and 548 nm. It was not possible to identify the type of the d–d transition, due to a strong charge transfer (CT) band tailing from the UV-region to the visible region^51^. The magnetic moment of at 298 K the **Fe(DTHMN)** is 5.83 B.M., which is in line with the Fe(III) ion’s octahedral geometry and the existence of five unpaired electrons^52^.

**Table 3:** Electronic spectral data, magnetic moments, and molar conductivity data for the **DTHMN** ligand^33^ and its metal chelates.

Compound	Electronic spectral bands (nm) λ_max_ (nm) DMF/[Reflectance]			µ_eff_. (B.M.)	Conductance (Ω^−1^ mol^− 1^ cm^2^)
	π- π^*^	*n*-π^*^	Other transitions		
DTHMN	264 [285]	329, 371 [352,456]	------	------	------
Ni(DTHMN)	276 [295]	380,421 [352,466]	500	3.50	12
Co(DTHMN)	282 [287]	356,441 [354,468]	497, 564	4.96	46
Fe(DTHMN)	276 [289]	338,430 [347,472]	548	5.83	83

### Conductivity measurements

The conductance values of 1 × 10^− 3^ M solutions of metal chelate in DMF were recorded and tabulated in Table 3. The values fall at 12 and 46 Ω^−1^ cm^2^ mol^− 1^ for **Ni(DTHMN)** and **Co(DTHMN)** chelates, respectively, indicating neutrality (non-electrolytic character). Usually, the relatively high value in the solution means that DMF molecules partially replace some anions. On the other hand, **Fe(DTHMN)** is a 1:1 electrolyte, with a conductance value of 83 Ω^−1^ cm^2^ mol^− 1^. This result agrees with the nitrate anion’s poor coordinating capacity in contrast to the acetate anion’s great coordinating ability^53^.

### Thermogravimetric analysis (TGA)

**Fig. 2:**
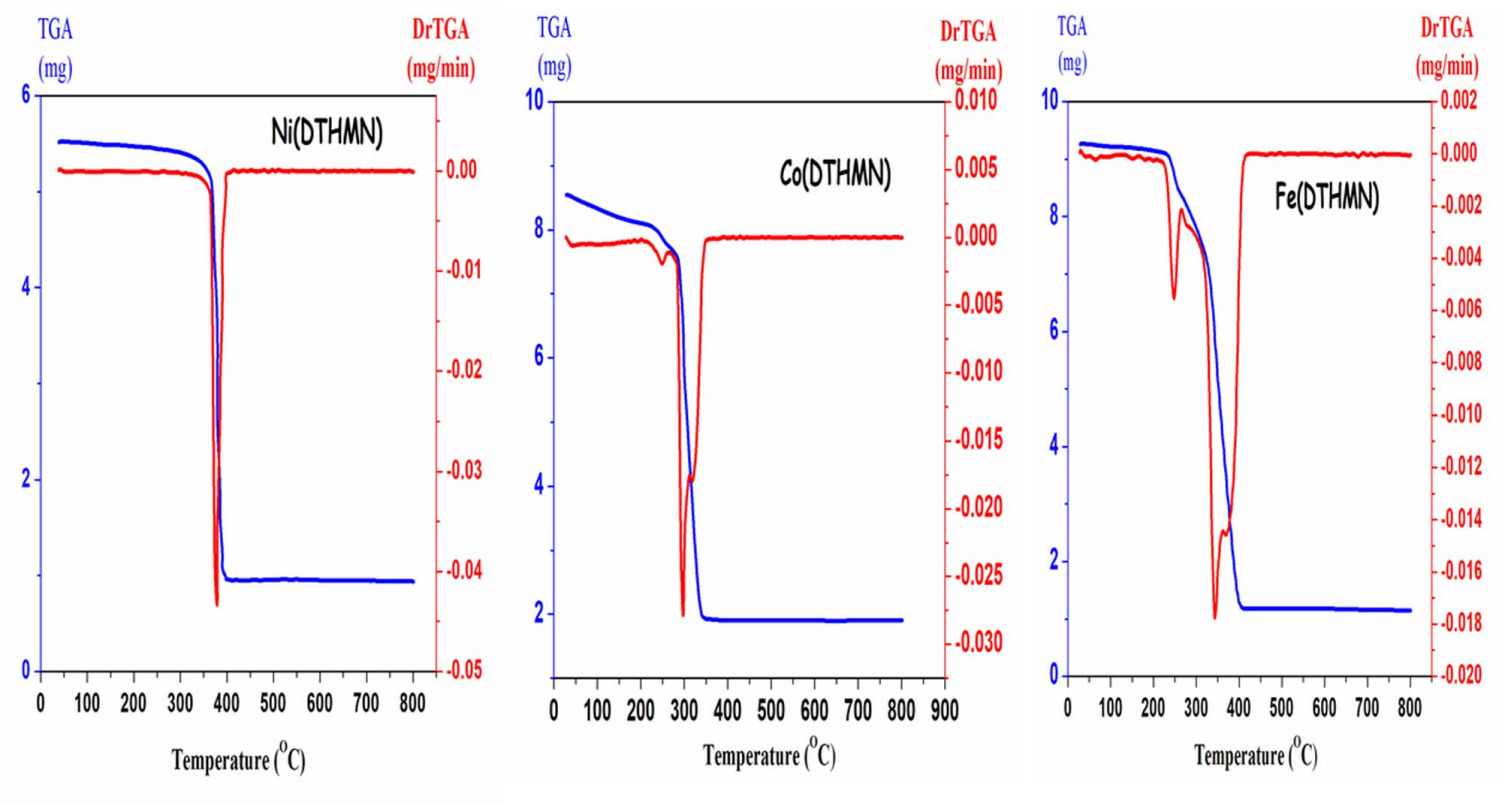
TGA-DTG curves of **M(DTHMN)** chelates.

Thermogravimetric analysis (TGA) is a technique used to investigate the thermal stability of a complex and to characterize the nature of solvent molecules, such as water, present in its solid state. Elemental analysis is perfectly complemented by thermal analysis. The decomposition step of [Ni(**DTHMN**)(OAc)] (**1**) (Fig. 2) presented elimination of CH_3_COOH, (C_6_H_5_)_2_, C_10_H_7_CN, (HCN)_2_, and N_2_; Found: 83.06% (Calcd: 83.68%). Finally, the possible residue is NiO + C; Found: 16.94% (Calcd: 16.23%), [Co(**DTHMN**)(OAc)(H_2_O)_2_].2H_2_O (**2**) (Fig. 2) showed three degradation stages at the temperature ranges: (28–175,176–269, and 270–420 °C). The first stage corresponds to a weight loss of two lattice water molecules; Found: 5.72% (Calcd: 5.94%). The second stage is related to the loss of two coordinate water molecules; Found: 5.11% (Calcd: 5.94%). The third stage at the range (270–420 °C) corresponds to the loss of CH_3_COOH, (C_6_H_5_)_2_, N_2,_ HCN, C_6_H_6,_ C_2_N_2_; Found: 66.90% (Calcd: 65.79%). Finally, the apparent residue is CoO + 5 C; Found: 22.27% (Calcd: 22.24%). [Fe(**DTHMN**)_2_]NO_3_ (**3**) (Fig. 2) exhibited two degradation stages at the temperature ranges: (28–270 °C, and 271–455 °C); The first stage corresponds to a weight loss of HNO_3,_ HCN molecules. Found: 9.48% (Calcd: 9.46%). The second stage is related to the loss of two C_10_H_8,_ (C_6_H_5_)_2_, C_6_H_5_CN, three C_2_N_2_, HCN, H_2_, half N_2_, and CO; Found: 78.34% (Calcd: 77.83%). Finally, the probable residue is FeO + 4 C; Found: 12.18% (Calcd: 12.61).

The Coats–Redfern method was utilized to obtain the kinetic and thermodynamic parameters of the chelates, as summarized in Table 4^54^. Additional thermodynamic activation parameters were determined using the Eyring equation. The following concludes the remarks: (i) The activation energy values of the chelates are in the range (17.41–385.2 KJ/mol). The order of E* values of chelates is **Ni(DTHMN**) > **Fe**(**DTHMN**) > **Co(DTHMN**) chelates, illustrating the greater thermal stability of **Ni**(**DTHMN**) than the other chelates, where E* depends on the (O-M-N) strength^55^. (ii) ΔH* values (14.42–379.77 kJmol^− 1^) are positive for all steps; this suggests that decomposition of these steps is endothermic^56^. (iii) The positive ΔS* values (0.0742–0.3288 Jmol^− 1^) denote that the triazine activated complex is less ordered than the reactants and/or the reactions are fast. On the other hand, the negative values (−0.0295 to −0.152 Jmol^− 1^) indicate that the reactants are less ordered than the activated complex and/or the reactions are slow. (iv) The positive values of ΔG* (56.34–165.43 kJmol^− 1^) illustrate the autocatalytic action of metal ions on thermal decomposition of the triazine chelates and nonspontaneous processes. In the first stage, the activation energy, enthalpy and entropy are the highest for **Ni(DTHMN**) and the lowest for **Co(DTHMN**).

**Table 4:** Temperatures of decomposition and the kinetic parameters of metal chelates.

No.	Step	*N* Order	T (K)	A (S^− 1^)	Δ E (kJ mol^− 1^)	ΔH (kJ mol^− 1^)	ΔS (kJ mol^1^ K^− 1^)	ΔG (kJ mol^− 1^)
(1)	First	1	652	5.57 × 10^30^	385.2	379.77	0.3288	165.43
(2)	First	0.66	360	1.68 × 10^7^	17.41	14.42	−0.1165	56.34
	Second	0.33	523	4.9 × 10^6^	33.40	29.06	−0.1297	96.84
	Third	1	570	9.27 × 10^11^	137.90	133.16	−0.0295	149.98
(3)	First	1	521	2.23 × 10^17^	175.44	171.11	0.0742	132.46
	Second	0.33	616	3.98 × 10^5^	61.08	55.96	−0.152	149.61

From the above findings, we could conclude that the elemental and thermal analyses, together with IR, UV/Vis, and magnetic measurements, confirm that the **Ni**(**DTHMN**) chelate has a tetrahedral structure, while the **Co**(**DTHMN**) and **Fe**(**DTHMN**) chelates exhibit octahedral geometries. These results agree with the conductivity data, indicating that the chelates are stabilized by strong coordination between the metal centers, the oxygen and nitrogen atoms of the **DTHMN** ligand.

### Crystal structure and morphological studies

#### XRD analysis

**Fig. 3:**
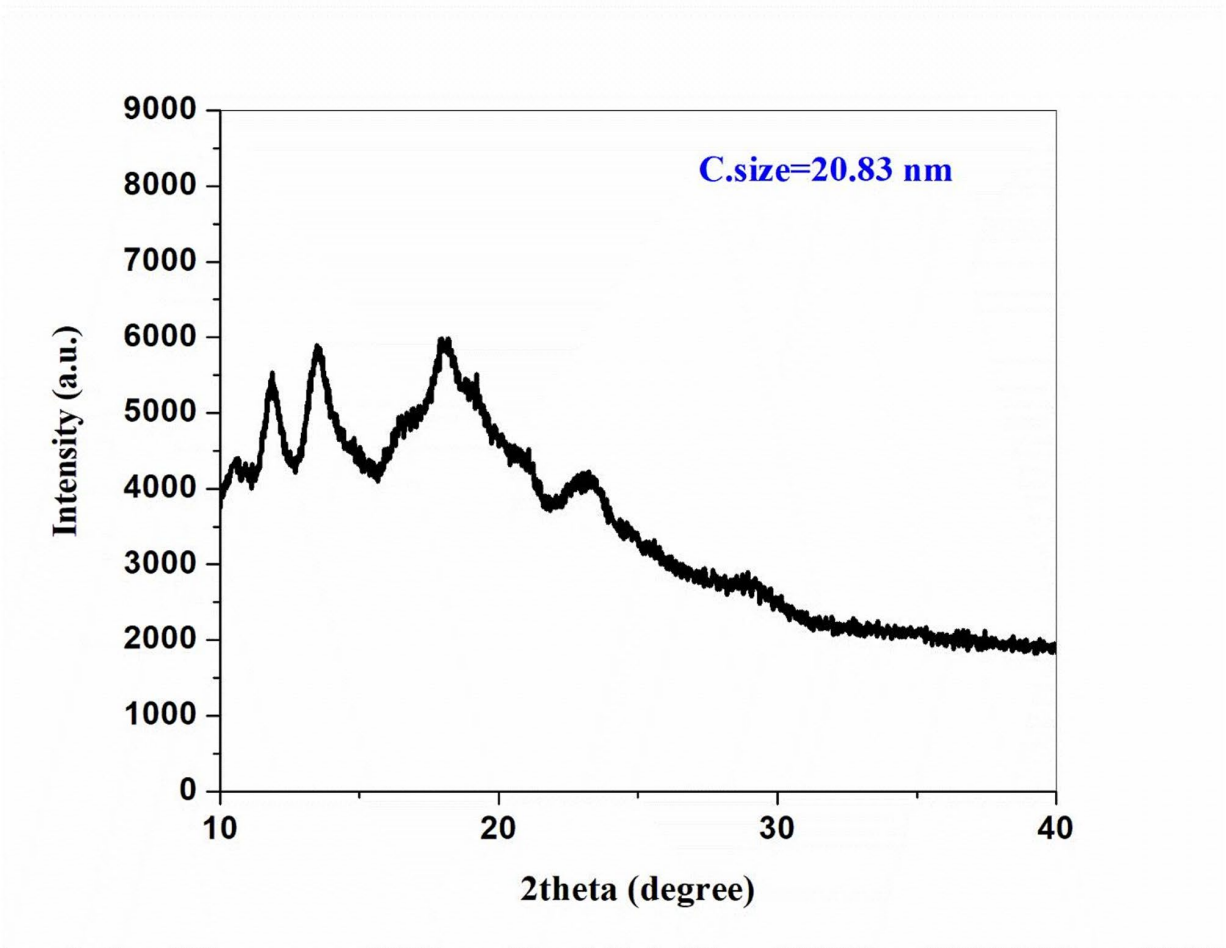
XRD pattern **Ni(DTHMN)** chelate.

The XRD patterns of the **M(DTHMN)** chelates indicate that these chelates possess a partially ordered structure, lying between amorphous and crystalline phases. The **Ni(DTHMN)** chelate (Fig. 3), along with its **Co(DTHMN)** and **Fe(DTHMN)** analogs, displays semicrystalline characteristics. The distinct shifts in diffraction angles and variations in peak intensities, relative to the free **DTHMN** ligand, provide clear evidence of complex formation through coordination between the metal centers and the donor sites of **DTHMN**^33^. Furthermore, by applying the Scherrer equation^57^ to the most prominent diffraction peaks, the estimated average crystallite sizes for **Ni(DTHMN)**, **Co(DTHMN)**, and **Fe(DTHMN)** were found to be approximately 21 nm, 17 nm, and 13 nm, respectively, confirming their nanoscale dimensions.

Figure 3.

#### TEM analysis

**Fig. 4:**
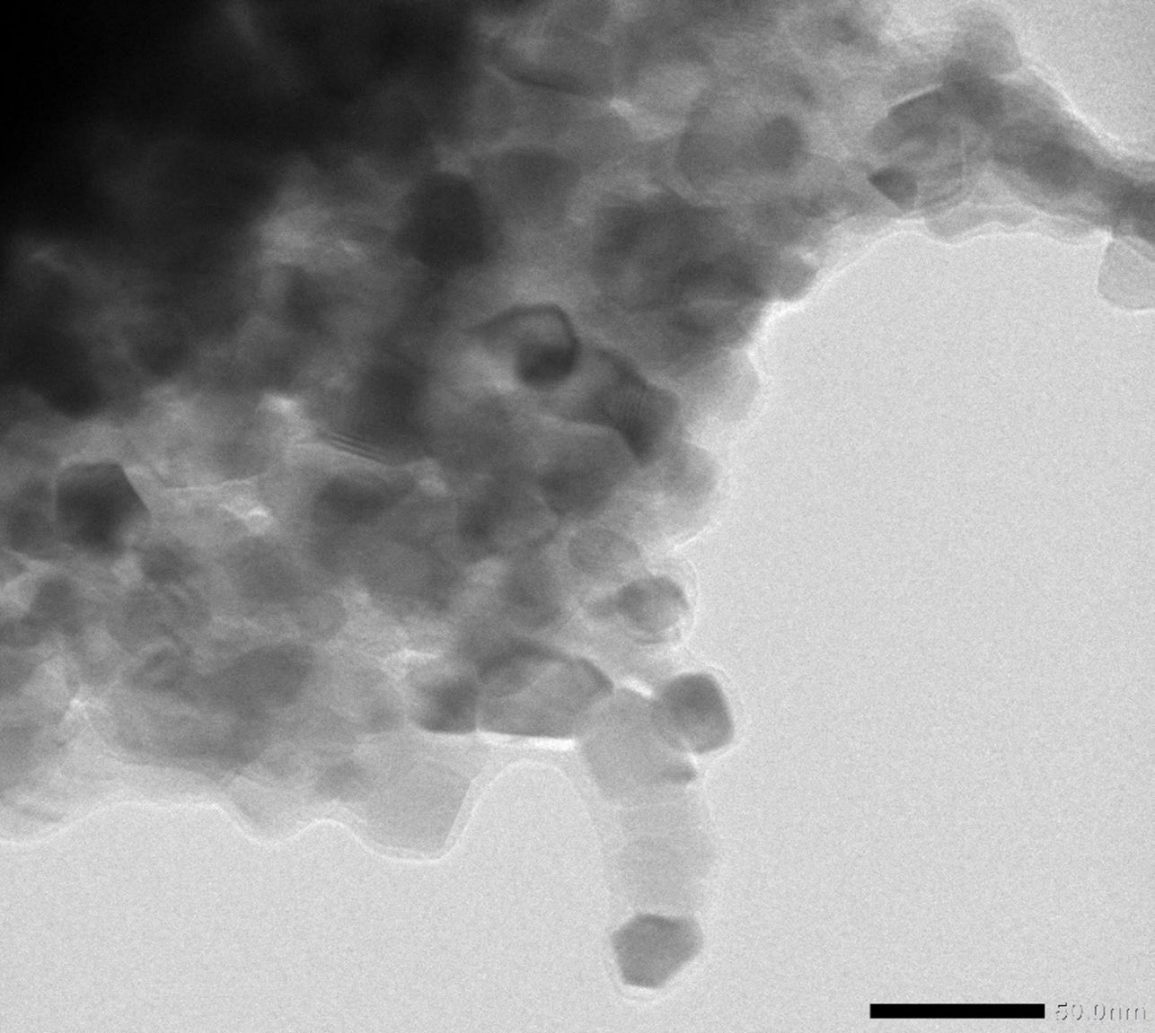
TEM image of **Ni(DTHMN)** chelate.

The morphology and particle size of the metal complexes’ particles were ascertained by TEM analysis. TEM images of **Ni(DTHMN)** are displayed in Fig. 4. The TEM picture of **Ni(DTHMN)** has a sphere and cubic shape with an average diameter of 25 nm. It has been established that the particle size of **Ni(DTHMN)** is nanoscale. The estimated crystallite size value derived from XRD analysis shows good agreement.

## Fluorescence spectra

### Effect of solvents on absorption and fluorescence spectra

**Table 5:** Solvatochromic data of the **M(DTHMN)** chelates.

Solvent	Ni(DTHMN) λ_ex_ = 335/nm		Co(DTHMN) λ_ex_ = 297/nm				Fe(DTHMN) λ_ex_ = 339/nm			
	λ_em_	(ν_a_-ν_f_)/cm^− 1^	(ν_a_ + ν_f_)/2	λ_em_	(ν_a_-ν_f_)/cm^− 1^	(ν_a_ + ν_f_)/2		λ_em_	(ν_a_-_νf_)/cm^− 1^	(ν_a_ + ν_f_)/2
**Chloroform**	514	10395.49	24653.00	402	8794.41	29272.83		444	6976.00	26010.52
**Ethylacetate**	511	10281.27	24710.11	377	7144.84	30097.62		354	1249.94	28873.56
**Benzene**	387	4010.95	27845.27	341	4344.52	31497.77		387	3658.73	27669.16
**1**,**4-Dioxane**	513	10357.57	24671.96	379	7284.81	30027.63		408	4988.72	27004.16
**Isopropanol**	519	10582.92	24559.28	333	3640.00	31850.03		483	8794.59	25101.23
**Ethanol**	523	10730.29	24485.60	372	6788.31	30275.88		495	9296.50	24850.27
**Methanol**	468	8483.23	25609.13	378	7215.01	30062.53		506	9735.68	24630.69
**DMF**	521	10656.89	24522.30	426	10195.86	28572.11		530	10630.60	24183.22
**Acetone**	500	9850.75	24925.37	382	7492.02	29924.02		351	1008.50	28994.28
**Toluene**	416	5812.29	26944.60	404	8917.56	29211.25		420	5689.00	26654.02

Several solvents with different polarities were used to examine the fluorescence spectra of the **DTHMN** ligand^33^ and its metal chelates. According to fluorescence spectra, the red or bathochromic shift is represented by the π π* transition. As solvent polarity increases, the excited state becomes more polar than the ground state^58,59^. The significant amplitude of Stoke’s shift suggests that the geometry of the excited state and the ground state may differ. As solvent polarity increases, it is generally observed that Stoke’s shift increases as well, indicating an increase in the dipole moment on excitation. Suppose the solute’s excited state charge distribution differs significantly from the ground state charge distribution and is such that it provides a greater interaction with polar solvents in the excited state. In that case, there may be a shift in the fluorescence wavelengths towards longer wavelengths^60^. Using the appropriate equations, the values of Lippert’s, Bakhshiev’s, and Kawski-Chamma-Viallet’s polarity functions were determined from the dielectric constant and refractive index and are provided in Table S1. In addition, the microscopic solvent polarity parameter (E_T_^N^) is presented in decreasing order of dielectric constants. Table 5 shows the arithmetic mean and solvatochromic shifts in various solvents derived from the absorption maxima (λ_ex_), fluorescence maxima (λ_em_)^61^, absorption wave number (ῡ_a_), and emission wave number (ῡ_f_). These data indicate that the fluorescence maxima (λ_em_) for **Ni(DTHMN**), **Co(DTHMN**), and **Fe(DTHMN**) chelates are 387–523 nm, 333–426 nm, and 351–530 nm, respectively. The interaction between the solute and solvent or the influence of the surrounding medium could be the reason for an increase in fluorescence maxima. Additionally, there are Stoke shifts for all the solvents used, which are in the range 4010–10,730 cm^−1^, 3640–10,195 cm^−1^ and 1008.5–10,630 cm^−1^ for **Ni(DTHMN)**, **Co(DTHMN)**, and **Fe(DTHMN)** chelates, respectively. Larger Stokes shifts indicate higher charge transfer.

### Estimation of ground and excited state dipole moments

The Lippert-Mataga, Bakhshiev, Kawski-Chamma-Vallet, and Reichardt equations were used to compute the dipole moments associated with the first excited singlet states of the current chelates. Figs. S1 and S2 depict the plots of Bakhshiev’s and Lippert’s polarity functions against Stokes shift (ῡ_a_ - ῡ_f_) for **Fe**(**DTHMN)** chelate. Fig. S3 displays Kawski-Chamma-Viallet’s polarity function against the arithmetic mean of the Stokes shift (ῡ_a_ + ῡ_f_)/2 for **Fe(DTHMN**) chelate. These graphs are fitted to a straight line, and the appropriate equations were used to estimate the ground and excited state dipole moments from the slopes. In Fig. S4, the microscopic solvent polarity parameter (E_T_^N^) is plotted against the Stokes shift (ῡ_a_ - ῡ_f_) for **Fe(DTHMN)** chelate, and the estimated change in dipole moment is displayed^62,63^. Table S2 provides the values of slopes, correlation coefficients, and the number of fitted data points. Correlation coefficients larger than R^2^ = 0.99 indicate that these correlations are linear. Table 6 provides the radius, dipole moment in the ground state, excited state, and change in dipole moments. The J T Edward method is used to determine the compound’s radius. Additionally, Table 6 shows that the dipole moment values of all the compounds produced experimentally are significantly higher in the excited state than in the ground state. Various solvent correlation methods exhibit differences in excited-state dipole moment values, which can be attributed to the assumptions adopted by those techniques. A high value of µ_e_ suggests that a molecule’s emission may come from a state that is more polar than the ground state and may also be caused by the excited state’s twisted intramolecular charge transfer (TICT)^64^. In the excited state, the dipole moment is increased by the transition π π*. This demonstrates that the compound contains an extensive π* electronic delocalized system with significant charged resonance structures in the excited state^65–69^. The following values were obtained by calculating the angles between the ground and excited state dipole moments: 120.15°, 120.68°and 121.31° for **Ni(DTHMN)**, **Co(DTHMN)** and **Fe(DTHMN**) chelates, respectively. These angles demonstrate that the dipole moments of the ground and excited states are not parallel to one another^70,71^.

**Table 6:** The dipole moments for **DTHMN** ligand^33^ and metal chelates in the ground (µ_g_) and excited (µ_e_) states (in Debye, D).

Compound	A	µ_g_^a^	µ_e_^a^	µ_g_^b^	µ_e_^b^	µ_g_^c^	µ_e_^c^	Δµ^d^	Cos φ	φ^o^
DTHMN	7.55	5.83	6.13	3.82	23.67	5.71	17.59	13.48	−0.838	147.02
Ni(DTHMN)	8.50	9.31	9.42	2.93	5.89	3.98	9.09	16.55	−0.502	120.15
Co(DTHMN)	8.82	6.25	9.17	8.29	16.93	4.84	19.64	15.1	−0.51	120.68
Fe(DTHMN)	8.20	3.75	5.96	9.11	18.96	2.00	18.64	11.51	−0.519	121.31

^a^ *Gaussian 09* using DFT software used to estimate the ground and excited states.

^b^ Calculated using (F_1_, F_2_) equations.

^c^ Calculated using (F_2_, F_3_) equations.

^d^ Calculated from E_T_^N^.

Lastly, there are a few explanations for the discrepancy between the experimental results of solvatochromic shift techniques and the theoretical data derived from TD-DFT results. Among these are: (i) Dependence on Onsager’s theory, which describes non-specific electrostatic interactions between solvents and solutes; (ii) disappearing of intermolecular H-bonds; (iii) the idea that the spherical Onsager radius causes the dipole moment in the Onsager cavity to remain constant; and finally (iv) as previously stated, an approximate co-linearity of the dipole moments in the ground and excited states^72^. However, DFT only yields dipole moment values for molecules in the gas phase. Several scholarly publications have documented a limited degree of concordance between theoretical and experimental values^73^.

## Antimicrobial activity

### Inhibition zone’s diameter determination

The in vitro antibacterial activity of the **DTHMN** ligand and its metal chelates was assessed using an agar disc against a variety of susceptible pathogens, such as *Staphylococcus aureus* as Gram-positive bacteria, *Escherichia coli* as Gram-negative bacteria, and *Candida albicans* as a fungus. The impact of various metal ions on the antibacterial activity was examined using a variety of metal chelates of **Ni(DTHMN)**, **Co(DTHMN)**, and **Fe(DTHMN)**. Table 7 showed that the **DTHMN** ligand had limited efficiency against all the bacterial strains that were studied, while most of the metal chelates were more active than the free ligand. This may be explained by Overton’s concept and chelation theory^74^. According to the results, **Ni(DTHMN)** chelate exhibits high activity against all sensitive bacteria, except for Candida albicans, which exhibits moderate activity. Furthermore, when compared to the reference control, **Fe(DTHMN)** chelate shows significant activity towards the sensitive microorganisms, while **Co(DTHMN)** chelate shows poor activity towards the same microorganisms. Metal ion complexes are absorbed by microorganisms into their cell walls, impairing their breathing and preventing them from producing the proteins needed for further growth. Thus, using metal ion complexes as antimicrobial agents is essential to preventing the growth of microorganisms^75^.

**Table 7:** Antibacterial activity of the ligand and **M(DTHMN)** chelates.

Average (mm) zone diameter						
Gram + bacteria			Gram – bacteria		Fungi	
S. aureus			E. coli		C. albicans	
No.	1000 µg/ml	500 µg/ml	1000 µg/ml	500 µg/ml	1000 µg/ml	500 µg/ml
DTHMN^33^	10 L	7 L	11 L	8 L	9 L	7 L
(1)	26 H	19 H	28 H	20 H	17 I	13 I
(2)	11 L	9 L	9 L	7 L	10 L	7 L
(3)	30 H	22 H	28 H	21 H	28 H	20 H
S	35	26	38	27	35	28

**S**: Standard drug such as Chloramphenicol in the case of Gram-positive bacteria, Cephalothin in the case of Gram-negative bacteria and cycloheximide in the case of fungi.

. The observed antimicrobial activity may be the result of the metal ion’s superior coordination with the chelating **DTHMN** ligand, which overlapped the ligand orbital and partially shared the ion’s positive charge with the donor groups. This reduced the metal ion’s polarity. Consequently, it increases the lipophilicity of the metal ion. This increases the chelates’ capacity to cross lipid membranes, deactivating the bonding sites of the microorganisms’ enzymes^76,77^. Coordination with the metal ions improved the effectiveness of the antimicrobial screening results. According to chelation theory, this type of higher efficiency can be demonstrated^78^. The produced compounds exhibited moderate activity for most of the tested strains. The following correlations were found between the biological results shown in Table 7 and the structural parameters data (*vide infra)* and/or IR spectral data in Table 2. The linear relationships between the biological activity against *E. coli* (*G-1*) *versus* the stretching frequency (Δν_C= N_) are observed as *G-1* = 0.2897 + 0.0281, Δν_C=N_/cm^− 1^, *r* = 0.96, *n* = 3 points except **Co(DTHMN)** chelate. The positive slopes imply that biological activity is increased as ν_C=N_ shifts to lower frequency, indicating a stronger bond between the metal ion and C = N.

2) The findings show that a greater degree of binding strength is associated with an increase in biological activity. This conclusion was supported by the positive slope of the linear correlations of the dipole moment against the biological data in Table 7 as follows: *G + 1* = −0.5213 + 0.1353µ/D, *r* = 0.99, *n* = 3 points; and *G-1*= −0.6175 + 0.1451 µ/D, *r* = 0.98, *n* = 3 points except **Fe(DTHMN)** chelate.

### MIC determination

A series of concentrations of a potent antimicrobial chelating agent was evaluated for their inhibitory effects against *Staphylococcus aureus*, *Escherichia coli*, and *Candida albicans* to determine their Minimum Inhibitory Concentrations (MICs)^35^. The MIC was defined as the lowest concentration at which no visible microbial growth was observed. Among the tested metal chelates, **Fe(DTHMN)** exhibited the most pronounced antibacterial activity against *E. coli*, with an MIC of 38 µg/mL surpassing its previously reported value of 41 µg/mL. Against *S. aureus*, both **Fe(DTHMN)** and **Ni(DTHMN)** demonstrated notable antimicrobial potency, with MICs of 71 µg/mL and 82 µg/mL, respectively. In the case of *C. albicans*, all evaluated chelates displayed marked antifungal activity, with **Fe(DTHMN)** and **Ni(DTHMN)** achieving MIC values of 37 µg/mL and 39 µg/mL, respectively.

## Theoretical studies

### DFT calculation

#### Frontier molecular orbitals (FMOs) analysis

**Fig. 5:**
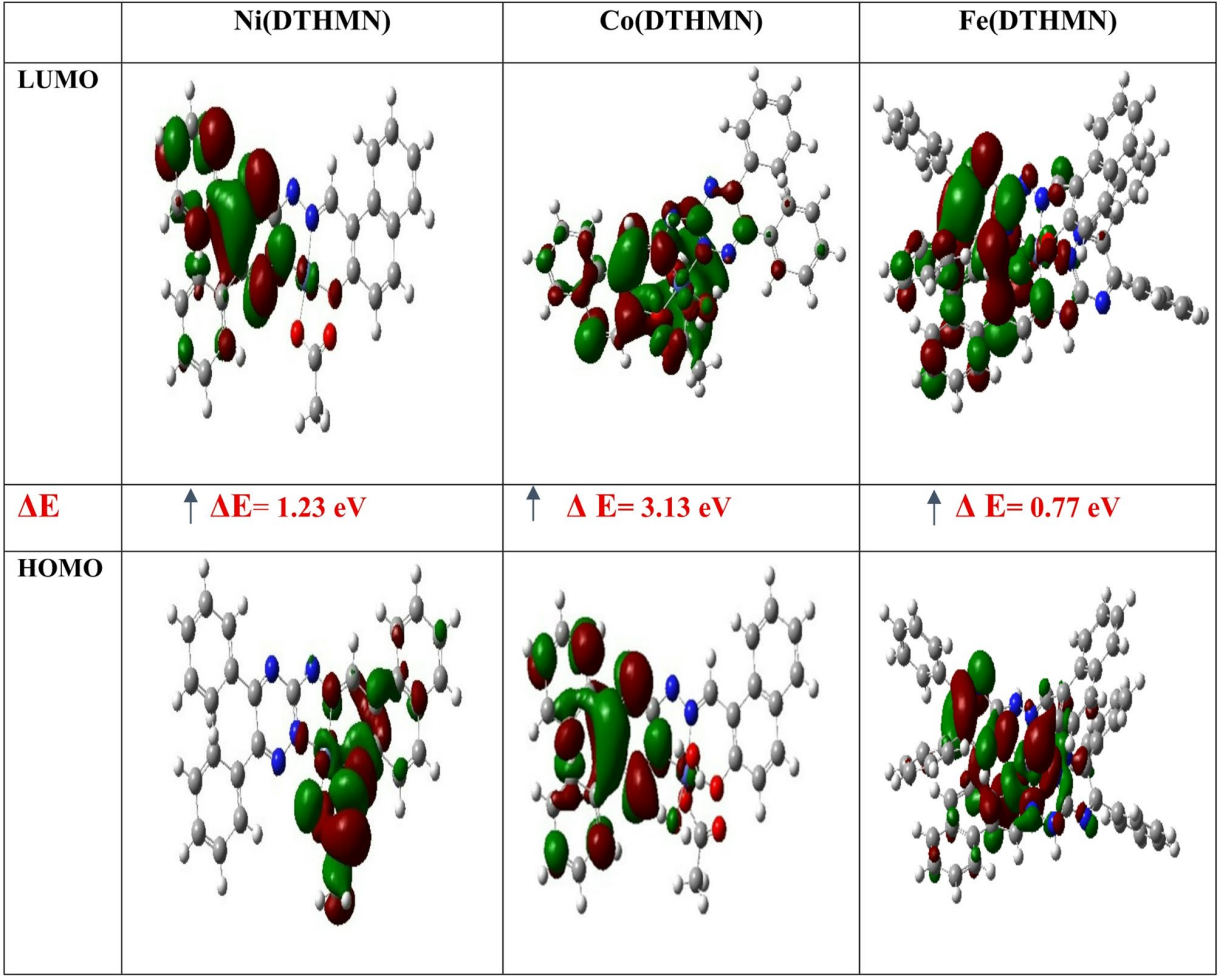
Graphical representation of HOMO-LUMO distribution of **M(DTHMN)**.

Using DFT based on the B3LYP/GENECP level with 6-311G(p, d) basis set for H, C, N, and O atoms and the SDD basis set for metal atoms implemented on *Gaussian 09*, the investigated compounds were optimized. The chemical reactivity, optical polarizability, and chemical hardness-softness that determine a molecule’s capacity for charge transfer can be obtained from the frontier molecular orbitals (FMOs), such as the highest occupied molecular orbital (HOMO) and lowest unoccupied molecular orbital (LUMO)^39^. Figure 5 displays the HOMO and LUMO energy levels of the produced compounds. For **Ni(DTHMN)** chelate, the HOMO contour showed that the electron density is localized over the N-triazine ring, N-hydrazino, acetate group and the part of 2-hydroxynaphthaldehyde, while the LUMO contour is more likely concentrated on the triazine and phenyl rings. Moreover, the energy level of the HOMO and LUMO of **Co(DTHMN)** indicates that the electron density is localized over the triazine ring and the majority of the phenyl rings, while for **Fe(DTHMN**) chelate, the electron density is concentrated over two triazine rings, side chains, 2-hydroxynaphthaldehyde moieties and part of the phenyl rings. On the other hand, the LUMO of **Ni(DTHMN)** is concentrated over triazine rings and phenyl rings. Whereas, for **Co(DTHMN)**, the LUMO orbital is localized over the triazine ring, side chain, 2-hydroxynapthaldehyde, part of the phenyl rings, the acetate group and water molecules. For **Fe(DTHMN)**, it is localized on one triazine ring, one phenyl ring, one of the two 2-hydroxynaphthaldehyde, and side chains.

#### Quantum chemical reactivity descriptors

Many fundamental chemical concepts are determined by the global chemical reactivity descriptors, which were computed using HOMO and LUMO energies. These include chemical potential (µ), electronegativity (χ), hardness (η), softness (S), and electrophilicity index (ω)^79–81^. Hardness (η) and softness (S), which are defined as (η = (E_LUMO_ – E_HOMO_)/2) and (S = 1/2η), respectively, are helpful ideas for comprehending the behavior of chemical systems. Using the electronic chemical potential and chemical hardness (ω = µ^2^/2η)^82^, the electrophilicity (ω) can be computed and incorporated into Table 8. Since the reactivity is inversely proportional to hardness, as shown in Table 8, the most reactive chelate is **Fe(DTHMN)**, which has the lowest E_gap_ (0.77 eV) and the lowest hardness (0.38 eV). In contrast, **Co(DTHMN)**, which is the less reactive compound, has the highest E_gap_ (3.13 eV) and the highest hardness (1.56 eV)^83^. Electronegativity values (χ/eV), ranging from 3.25 to 3.97 eV, represent a crucial property of the compounds under study (as shown in Table 8). Notably, the **Fe(DTHMN)** chelate exhibits the lowest electronegativity value, whereas the **Co(DTHMN)** chelate displays a higher value. According to ω/eV values, which help relate a molecule’s electron-donating capacities^84^, the **Co(DTHMN)** chelate is the greatest electrophile, while the **Fe(DTHMN)** chelate is the poorest electrophile. Lastly, in most chelates, the bond lengths of the **DTHMN** ligand in the vicinity of coordinating centers are generally more elongated in the following ranges: C_8_ = N_7 azomethine_ (0.048–0.059), C_11_-O_26_ (0.05–0.09), and C_2_ = N_1 triazine_ (0.01–0.06). Additionally, it was noted that the C_8_ = N_7 azomethine_ bond length is longer than the free ligand in all metal chelates. This could be explained by the C_8_ = N_7_ azomethine’s double bond character being reduced when it coordinates with the metal ions. This is consistent with the C_8_ = N_7 azomethine_ absorption bond values in the free **DTHMN** and all metal chelates (Table S3). When compared to the free **DTHMN**, it was demonstrated that the C_8_ = N_7 azomethine_ in all metal chelates was displaced to lower frequencies.

**Table 8:** Molecular Structural parameter for the free ligand and metal chelates using B3LYP/GENECP with 6-311G (d, p).

Compound No.	E_T_,au kcal/mol	E_HOMO_ (eV)	E_LUMO_ (eV)	E_gap_ (eV)	Softness S(eV^− 1^)	Hardness ƞ(eV)	Electronegativity χ (eV)	Electrophilicity ω (eV)	µ = E_LUM_+ E_HOMO_/2	µ,Ɗ
**DTHMN**	−1351	−5.43	−1.96	3.47	0.576	1.735	3.69	3.93	3.69	5.83
**(1)**	−1748	−4.26	−3.03	1.23	1.624	0.616	3.64	10.76	−3.64	9.31
(**2)**	−2477	−5.54	−2.41	3.13	0.638	1.565	3.97	5.03	−3.97	6.25
**(3)**	−3371	−3.64	−2.87	0.77	2.597	0.385	3.255	13.76	−3.26	3.75

#### MEP analysis

**Fig. 6:**
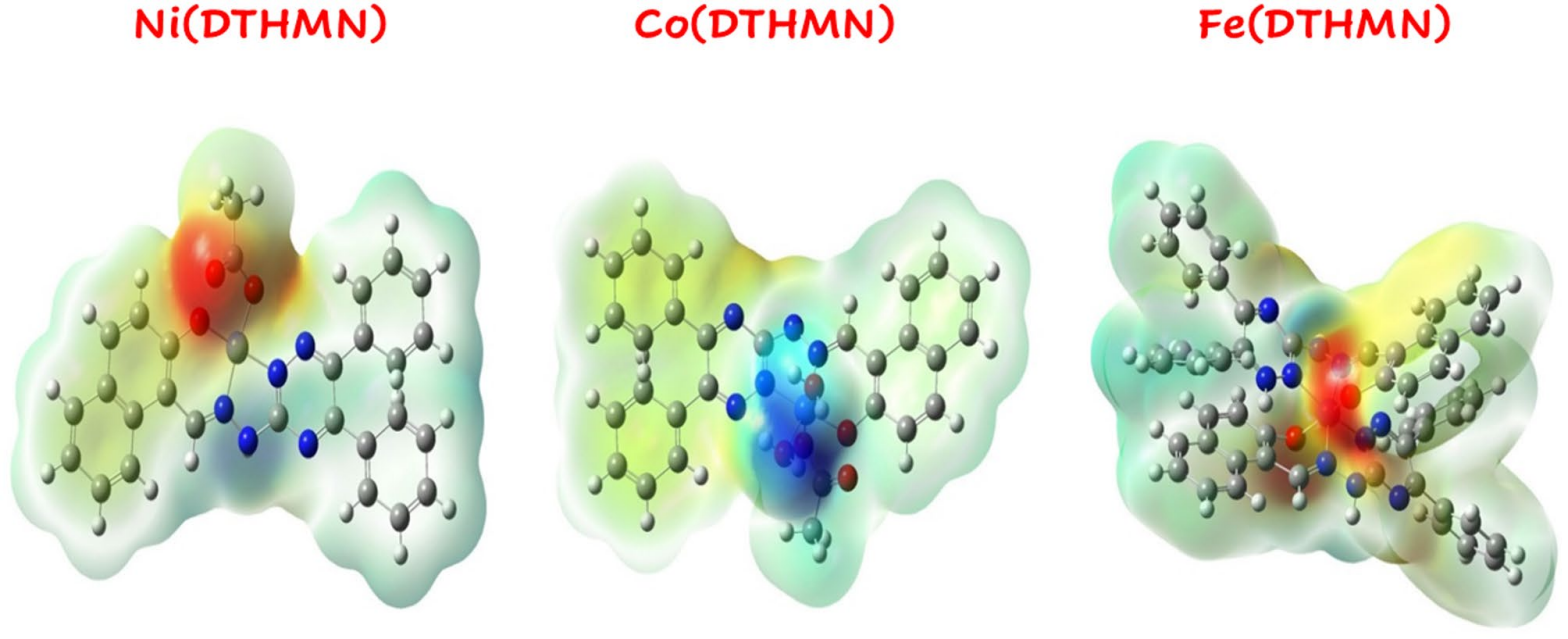
MEP diagram from Alpha SCF density of **M(DTHMN)** chelates.

Diagrams of a molecule’s electrostatic potential (MEP) show how charges are distributed throughout it and provide details on its size, composition, and value. The optimum chemical locations for interacting with other molecules can be predicted using this method. Figure 6 illustrates that in 3D MEP maps, the red color denotes electron-rich regions while the blue color denotes poor regions of the molecule^85^. For the **DTHMN** ligand, an electronegative region is present over triazine nitrogen, azomethine nitrogen, and phenolic oxygen, confirming that they are chelating sites^33^. Also, for all metal chelates, it is shown that the maximum electronegative region is localized over triazine nitrogen, azomethine nitrogen and phenolic oxygen, while the maximum electropositive region is concentrated over the side chain for **Ni(DTHMN)** and **Fe(DTHMN)** chelates and over the coordinated water molecule for **Co(DTHMN)** chelate.

#### Nonlinear optical (NLO) study using DFT

Essential components like frequency shifting, optical modulation, switching, lasers, fiber, optical materials, and optical memory are NLO characteristics for developing technologies such as signal processing, telecommunications, and optical interconnections^86,87^. DFT computational analysis was used to investigate the relationship between molecular structure and NLO properties^80^. The equations evaluated the total static dipole moment (µ_tot_), polarizability anisotropy (α), the mean polarizability (Δα), and the overall first hyperpolarizability (β_tot_)^88,89^. The produced chelates have readily polarizable electrons due to donor, acceptor, and conjugation links, which is advantageous for NLO active characteristics^90^. The energy gap between HOMO and LUMO levels is stabilized, depending on polarization during the electric field absorption, which can be measured by the dipole moment^91^. The calculated static polarizability and hyperpolarizability values for the **DTHMN** ligand and its metal chelates are shown in Tables 9 and 10. Metal chelates are found to have values between 13.02 and 77.03 atomic units when the concept of linear polarizability is examined. Notably, the **Fe(DTHMN)** chelate exhibits the maximum degree of polarizability. Additionally, β values for the chelates range from 111.15 to 413.25 atomic units. It is noteworthy that the **Co(DTHMN)** chelate exhibits a higher β value of 413.25 a.u. The initial static hyperpolarizability values (β_tot_) of the chelates range from 0.96 × 10^− 30^ to 3.57 × 10^− 30^ (esu). Therefore, all the produced compounds exhibited significantly higher hyperpolarizability than the standard molecule for assessing NLO behavior, urea (β_tot_ = 43 a.u.)^92^. The **Ni(DTHMN)** chelate’s hyperpolarizability (1.79 × 10^− 30^ esu) is five times higher than that of urea (0.781 × 10^− 30^ esu). On the other hand, the **Fe(DTHMN)** chelate (0.96 × 10^− 30^ esu) is three times greater than urea, and the **Co(DTHMN)** chelate (3.57 × 10^− 30^ esu) is ten times greater than urea. Based on the urea-relative study, all the produced compounds are suitable candidates for NLO applications.

**Table 9:** The dipole moment (µ), the mean polarizability (α), the anisotropy of the polarizability (∆α) for the prepared compounds.

Compound No.	µ_x_	µ_y_	µ_z_	µ_total_	α_xx_	α_yy_	α_zz_	α_xy_	α_xz_	α_yz_	<α> (au)	<α> (esu) x10^− 23^	∆α (au)	∆α (esu) x10^− 24^
DTHMN	−1.51	5.20	2.16	5.83	149.65	162.13	197.52	10.86	5.40	8.81	169.74	2.51	48.46	7.18
(1)	7.12	−5.24	−2.91	9.31	160.99	203.28	231.59	3.73	7.07	−1.56	198.62	2.94	63.01	9.34
(2)	6.79	−4.43	4.28	9.17	232.01	216.96	228.17	3.83	1.51	−10.83	225.71	3.34	13.02	1.93
(3)	−3.16	0.23	−2.01	3.75	294.02	378.65	358.27	2.91	7.01	−15.60	343.65	5.09	77.03	11.41

**Table 10:** Calculated hyperpolarizability (β_tot_) components for the synthesized compounds.

Compound No.	β_xxx_	β_xyy_	β_xzz_	β_yyy_	β_yxx_	β_yzz_	β_zzz_	β_zyy_	β_total_ (au)	β_total_ (esu) x 10^− 30^
DTHMN	−68.59	−40.28	−0.079	−13.83	−16.21	3.25	9.31	−3.62	153.35	1.32
(1)	172.67	36.98	−5.88	34.35	−9.54	4.28	−16.72	−17.50	207.08	1.79
(2)	217.05	88.25	54.23	−134.82	−8.64	−52.72	23.61	−7.67	413.25	3.57
(3)	−129.63	−4.64	32.22	−34.36	5.54	2.25	−28.37	40.41	111.15	0.96

### Molecular docking

Molecular docking studies are essential for understanding how compounds interact with enzymes and predicting their binding conformations within active sites. These techniques are vital in computational drug design, as they assess the interaction dynamics between newly synthesized compounds and their enzyme targets. By identifying the optimal binding orientations and structural features that minimize the energy of the enzyme-ligand chelate, molecular docking provides a comprehensive analysis of these interactions^93^. This approach offers key insights into the potential efficacy and mechanisms of action of novel compounds, enabling the rational design and optimization of pharmacologically active agents, and improving the efficiency and success rate of drug discovery^94^.

**Fig. 7:**
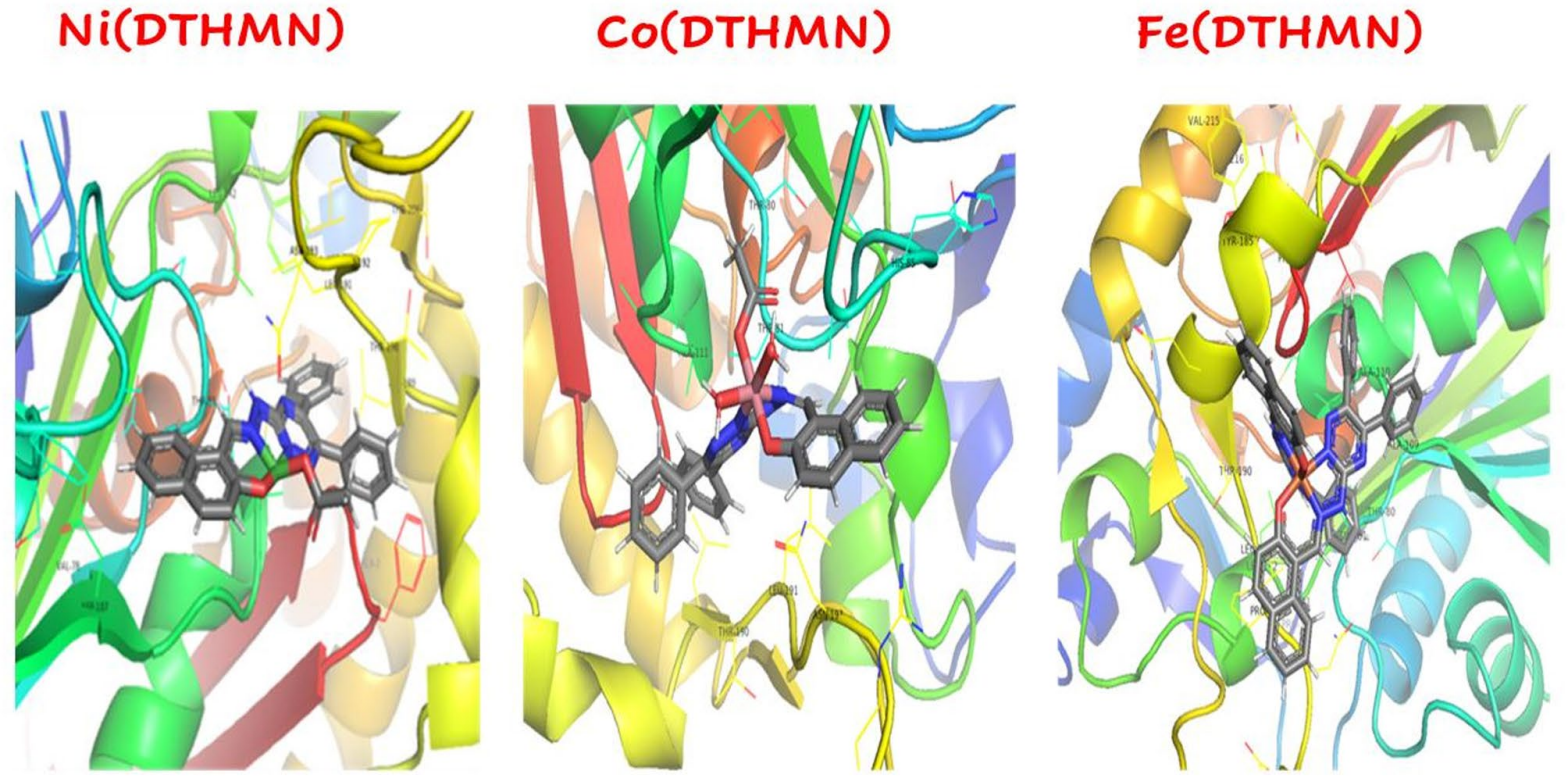
3D interactions of docked compounds **M**(**DTHMN)** chelates.

We initiated our molecular docking study by validating the docking approach through a re-docking process using the native ligand co-crystallized with the 1HNJ active site. This validation step was essential to confirm the reliability of our docking methodology for the current investigation. The re-docked ligand successfully reproduced the binding pattern of the co-crystallized ligand, demonstrating a close alignment with an RMSD (Root Mean Square Deviation) of 1.106 angstroms, as demonstrated in Fig. S5. According to the literature^95^ A scoring function is considered effective if the RMSD of the best-docked conformation of the native ligand is below 2.0 angstroms. Additionally, the hydrogen bonds formed between the docked ligand and the amino acids in the 1HNJ protein (ARG151, GLY209, THR28, ASN210, ASN247, ARG36, TRP32) closely resembled those formed by the native ligand. This strong similarity further supports the accuracy of our docking simulations, as shown in Fig. S5. The 3D interactions between various chelates and the target 1HNJ protein, as revealed through molecular docking studies, are depicted in Fig. 7. The **Ni(DTHMN)** chelate forms a hydrogen bond between nitrogen atom N16 and threonine)THR 81(, with a bond distance of 2.32 Å and a binding energy of − 8.0 kcal/mol, indicating a relatively strong interaction. In comparison, **Co(DTHMN)** interacts with threonine (THR 81) at a longer bond distance of 3.38 Å and exhibits a weaker binding energy of − 7.3 kcal/mol, suggesting a less stable interaction. **Fe(DTHMN)** demonstrates the most favorable interaction, forming a hydrogen bond between the nitrogen atom N59 and glycine)GLY 186(at a short distance of 2.27 Å, along with a binding energy of − 8.1 kcal/mol. The correlation between the molecular docking data and the antibacterial activity reveals that stronger hydrogen bonds and higher binding energies generally correspond to increased antibacterial potency. Specifically, **Fe(DTHMN)**, with the shortest bond distance (2.27 Å) and most negative binding energy (–8.1 kcal/mol), exhibits the highest antibacterial activity, indicated by a 21 mm inhibition zone. **Ni(DTHMN)**, also demonstrating strong binding characteristics (2.32 Å, − 8.0 kcal/mol), shows a slightly lower yet still significant antibacterial effect (20 mm inhibition zone). In contrast, **Co(DTHMN)**, with the longest bond distance and the least negative binding energy, corresponds to the weakest antibacterial activity, reflected in a minimal inhibition zone of 7 mm.

It is also worth noting that, although the docking results support the observed binding affinity trends, discrepancies between theoretical predictions and experimentally observed antimicrobial activities may be attributed to some biological factors such as cell permeability, efflux mechanisms, metabolic stability, or off-target interactions parameters that are not fully accounted for in docking simulations.

## Conclusion

In this study, three novel metal chelates, **Ni(DTHMN)**, **Co(DTHMN)**, and **Fe(DTHMN)**, were successfully synthesized using a tridentate ligand derived from fused triazine and 2-hydroxy-1-naphthaldehyde frameworks. Comprehensive characterization was conducted through elemental analysis, infrared (IR) and UV-Vis spectroscopy, thermogravimetric analysis (TGA), molar conductivity, and magnetic susceptibility measurements. Spectral and structural assessments confirmed that the **DTHMN** ligand functions as a monobasic tridentate (ONN) chelator, coordinating through the azomethine nitrogen, triazine nitrogen, and deprotonated phenolic oxygen atoms. The metal chelates exhibited both octahedral and tetrahedral geometries, depending on the nature of the central metal ion. Antimicrobial assays against a variety of bacterial and fungal strains revealed that the metal chelates displayed markedly superior bioactivity compared to the **DTHMN** ligand, highlighting the enhancement in biological efficacy upon complexation. The synthesized compounds also demonstrated pronounced solvatochromic behavior. Linear Solvation Energy Relationship (LSER) analysis, based on multiple solvent polarity parameters, indicated a bathochromic shift (red shift) in π–π* transitions with increasing solvent polarity. Solvatochromic data further enabled the estimation of dipole moments in the ground state (µ_g_) and the excited state (µ_e_), revealing that the excited state possesses a higher polarity and polarizability than the ground state. Density Functional Theory (DFT) computations were employed to optimize the geometry of the **DTHMN** ligand and its corresponding metal chelates. In addition to structural optimization, critical quantum chemical descriptors were calculated to further elucidate the electronic properties. Molecular docking studies were conducted to elucidate the binding and interaction modes of the newly synthesized bioactive compounds with the Escherichia coli receptor (PDB ID: 1HNJ). Among the tested chelates, **Fe(DTHMN)** exhibited the most potent binding affinity, with a binding energy of −8.1 kcal/mol, suggesting that **Fe(DTHMN)** may serve as a promising inhibitor of the 1HNJ protein. Future work will involve the synthesis and elucidation of new **DTHMN**–metal chelates with diverse transition metals to explore their coordination behavior and electronic structures. Advanced characterization and biological evaluation will be performed to clarify structure–activity relationships and identify promising candidates for biomedical applications.

## Supplementary Information

Below is the link to the electronic supplementary material.

Supplementary Material 1

## Data Availability

Yes, availability of Data and Materials. The datasets used and/or analyzed during the current study are available from the corresponding author on reasonable request.
