# Supplementary material for: Solvatochromic, spectroscopic, DFT calculations, antimicrobial and docking studies of new Fe(III), Co(II), and Ni(II) chelates containing 1,2,4-triazine
Source: Sci Rep. 2026 Apr 25;16:13406. doi: 10.1038/s41598-026-48416-3 (PMC13110366; doi:10.1038/s41598-026-48416-3)
Supplement: Supplementary file 1 — Supplementary Material 1 [file 41598_2026_48416_MOESM1_ESM.docx]

Table S1. Calculated values of solvent parameters (n, ε), microscopic solvent polarity (E_T_^N^) and solvent polarity functions (F_1_, F_2_, F_3_), solvents are listed in the order of increasing ε.

| **Solvent** | **Ε** | **N** | **E_T_^N^** | **F_1_(ε,n)** | **F_2_(ε,n)** | **F_3_(ε,n)** |
| --- | --- | --- | --- | --- | --- | --- |
| **1,4 Dioxane** | 2.25 | 1.4224 | 0.1636 | 0.0245 | 0.0499 | 0.290 |
| **Benzene** | 2.28 | 1.5010 | 0.1110 | 0.003 | 0.0040 | 0.300 |
| **Chloroform** | 4.81 | 1.4490 | 0.2593 | 0.1483 | 0.3709 | 0.825 |
| **Ethylacetate** | 6.02 | 1.3724 | 0.2284 | 0.1996 | 0.4891 | 0.490 |
| **Isopropanol** | 17.80 | 1.3993 | 0.5864 | 0.2641 | 0.7532 | 0.650 |
| **Acetone** | 20.70 | 1.3588 | 0.3548 | 0.2843 | 0.7903 | 0.654 |
| **Ethanol** | 24.50 | 1.3614 | 0.6543 | 0.2887 | 0.8127 | 0.924 |
| **Methanol** | 32.50 | 1.3284 | 0.7623 | 0.3086 | 0.8547 | 0.650 |
| **DMF** | 38.25 | 1.4300 | 0.4040 | 0.2750 | 0.8400 | 0.710 |
| **Toluene** | 2.38 | 1.4961 | 0.099 | 0.0132 | 0.0291 | 0.3499 |

Table S2. Intercept (C), the slope (S), correlation coefficient (r) and number of data points (n) corresponding to the statistical treatment of spectral shifts of metal complexes

| **Fe(DTHMN)** | | | | **Co(DTHMN)** | | | | **Ni(DTHMN**) | | | |  |
| --- | --- | --- | --- | --- | --- | --- | --- | --- | --- | --- | --- | --- |
| n | *r* | S | C | n | *r* | S | C | n | *r* | S | C |  |
| 6 | 0.99 | 14476 | 5031.5 | 5 | 0.93 | 9376 | 4561.9 | 5 | 0.91 | 1279.1 | 10286 | **ν_stoke_ *vs* F_1_** |
| 6 | 0.99 | 5076 | 5135.6 | 5 | 0.90 | 3212 | 4699.5 | 5 | 0.93 | 429.6 | 10300 | **ν_stoke_ *vs* F_2_** |
| 8 | 0.98 | 6295 | 29295.0 | 5 | 0.89 | 5312 | 32585.0 | 5 | 0.96 | 1098.7 | 27321 | **(ν_a_+ν_f_ )/2 *vs* F_3_** |
| 7 | 0.94 | 7998 | 4061.9 | 6 | 0.88 | 11058 | 1818.6 | 5 | 0.93 | 14842.0 | 3702.6 | **ν_stoke_ *vs* E_T_^n^** |

| **No.** | **Charges** | | | | **Bond length** | | | | | |
| --- | --- | --- | --- | --- | --- | --- | --- | --- | --- | --- |
|  | **N_7_**  **Azomethine** | **N_1_**  **Triazine** | **O_26_**  **2-hydroxy naphthaldehyde** | **M** | **C_8_=N_7_**  **azomethine** | **M-O_26_** | **M-N_7_** | **M-N_1_** | **C_11_-O_26_** | **C_2_=N_1_**  **Triazine** |
| **DTHMN [33]** | -0.128 | -0.199 | -0.300 | ---- | 1.242 | ---- | ---- | ---- | 1.35 | 1.34 |
| **1** | -0.503 | -0.519 | -0.615 | 0.752 | 1.29 | 1.82 | 1.86 | 1.85 | 1.446 | 1.28 |
| **2** | -0.201 | -0.437 | -0.568 | 0.848 | 1.29 | 2.05 | 2.05 | 2.1 | 1.299 | 1.357 |
| **3** | -0.390 | -0.563 | -0.459 | 1.279 | 1.3015 | 1.805 | 1.886 | 1.8576 | 1.4318 | 1.3344 |

Table S3. The selected bond lengths and charge of the ligand and **M(DTHMN)** chelates

Fig. S1. The relation between Stoke shift/cm^-1^ *versus* F_L-M_ of [Fe(**DTHMN**)_2_]NO_3_

Fig. S2. The relation between Stoke shift/cm^-1^ *versus* F_B_ of [Fe(**DTHMN**)_2_]NO_3_

Fig. S3. The relation between average/cm^-1^ *versus* F_K-C-V_ of [Fe(**DTHMN**)_2_]NO_3_

Fig. S4. The relation between Stoke shift/cm^-1^ *versus* E_T_^N^ of [Fe(**DTHMN**)_2_]NO_3_

| 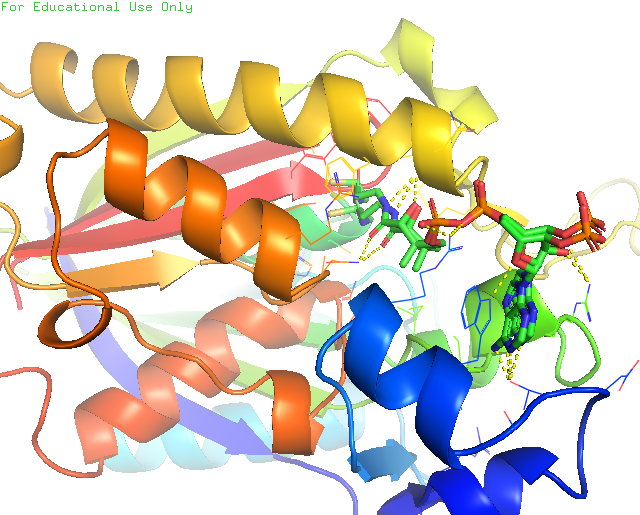 |
| --- |
| Fig. S5: 3D representation of the superimposition of the co-crystallized and the redocked ligand in 1HNJ. |

**Data S.1**

**Synthesis of 1-((2-(5,6-diphenyl-1,2,4-triazin-3-yl)hydrazono)methyl) naphthalen-2-ol (DTHMN)**

5,6-Diphenyl-3-hydrazino-1,2,4-triazine (HT) was prepared according to the method reported in the literature [29]. The hot ethanolic solution of HT was added to the acidified solution of 2-hydroxy-1-naphthaldehyde in a molar ratio of 1:1). The mixture was heated under reflux for 2h. The yellowish precipitate was filtered off and washed with hot ethanol; the dried product DTHMN has m.p. above 300 ^o^C with a yield 94%. The detailed synthetic steps for the DTHMN ligand are given in Scheme 1. **IR (KBr,ν, cm^-1^):** 1617 ν(C=N_azomethine_), 1518 ν(C=N_triazine_), 1464 ν(N=N_triazine_), 1293 ν(N–N_triazine_) **UV/Vis (DMF)**: 264, 329,372. **^1^H NMR (DMSO-d₆, δ, 300 MHz)**: 7.25(d, 1H, Ar-H), 7.40-7.63(m, 12H, Ar-H), 7.90 (d, 2H, Ar-H), 8.23 (d, 1H, Ar-H), 9.35 (s, 1H,12.30 (bs, 1H, NH exchangeable with D_2_O), 12.79 (bs, 1H, OH exchangeable with D_2_O). **Elemental Analysis**: Calculated for C_26_H_19_N_5_O (417.76): C, 74.75%; H, 4.58%; N, 16.76; Found: C, 74.16%; H, 4.58%; N, 16.99.

**Data S.2**

Elemental analyses for carbon, hydrogen, and nitrogen were performed using a Vario EL-Elementar analyzer at the Chemical War Department, Ministry of Defense. Metal ion content was determined following the decomposition of accurately weighed samples of the metal chalets with concentrated nitric acid. The resulting solutions were neutralized with ammonia and titrated using ethylenediaminetetraacetic acid (EDTA). Thermal decomposition temperatures were measured using a Stuart SMP3 melting point apparatus. Infrared (IR) spectra were recorded using a Nicolet IS10 FT-IR spectrometer. Proton nuclear magnetic resonance (^1^H-NMR) spectra were obtained at ambient temperature on a Bruker WP 200 SY spectrometer. UV-Vis absorption spectra in the 200–800 nm range were recorded in either reflectance or dimethylformamide (DMF) solutions using a Jasco V-550 spectrophotometer. Fluorescence emission data were collected at Ain Shams University, Cairo, using a PerkinElmer LS 55 luminescence spectrometer (USA). Molar conductivity measurements of 10⁻³ M solutions of the complexes in DMF were carried out using a Corning Model 441 conductivity meter (NY 14831). Magnetic susceptibility data were collected *via* the Gouy balance method at room temperature using a Sherwood Scientific magnetic susceptibility balance (Cambridge Science Park, England). The effective magnetic moments (μ_eff_) were calculated using the equation μ_eff_ = 2.828(χ_m·T)^½ B.M., where χm is the molar susceptibility corrected for diamagnetic contributions using Pascal’s constants. Electron spin resonance (ESR) spectra were recorded on a Bruker EMX X-band spectrometer. X-ray diffraction (XRD) patterns were obtained using a PHILIPS diffractometer equipped with CuKα₁ radiation (λ = 1.54056 Å), operated at 40 kV and 30 mA. Transmission electron microscopy (TEM) images were captured using a JEM-2100 instrument (JEOL) at an accelerating voltage of 200 kV. Thermogravimetric analysis (TGA) was conducted using a Shimadzu TGA-50 thermal analyzer, with samples heated from room temperature to 800 °C at a rate of 10 °C/min.
